# ﻿*Smicromyrmefrankburgeri* Schmid-Egger (Hymenoptera, Mutillidae), a replacement name for *S.burgeri* Schmid-Egger, 2021, preoccupied by *S.burgeri* Lelej, 2020

**DOI:** 10.3897/zookeys.1097.83215

**Published:** 2022-04-21

**Authors:** Christian Schmid-Egger, Stefan Schmidt

**Affiliations:** 1 Fischerstr. 1, 10317 Berlin, Germany Unaffiliated Berlin Germany; 2 SNSB-Zoologische Staatssammlung München, Munich, Germany SNSB-Zoologische Staatssammlung München Munich Germany

**Keywords:** Velvet ants, homonymy, replacement name

## Abstract

*Smicromyrmeburgeri* Schmid-Egger, 2021, a junior homonym of S. (Eremotilla) burgeri Lelej, 2020, is replaced with Smicromyrme (Smicromyrme) frankburgeri Schmid-Egger, **nom. nov.**

In a recent publication (Schmid-Egger and Schmidt 2021) a new species of Mutillidae from Germany was described as Smicromyrme (Smicromyrme) burgeri Schmid-Egger. The name of the new species turned out to be preoccupied by Smicromyrme (Eremotilla) burgeri Lelej, 2020, a species described from Karnataka, India (Lelej 2020), only one year before the German species was described. Both species were named after the German entomologist Frank Burger.

In accordance with Article 60 of the International Code of Zoological Nomenclature (International Commission on Zoological Nomenclature 1999), the junior homonym is replaced with a new name, Smicromyrme (Smicromyrme) frankburgeri Schmid-Egger, nom. nov.

*Smicromyrme* is a genus of velvet ants (Mutillidae) with currently approximately 270 described species from the Palearctic, Afrotropical, and Oriental regions (Pagliano et al. 2020). The Indian species, *S.burgeri* was described in the subgenus Eremotilla (Lelej 2020), whereas the German species belongs to the nominate subgenus.
